# Breast cancer risk after exposure to perfluorinated compounds in Danish women: a case–control study nested in the Danish National Birth Cohort

**DOI:** 10.1007/s10552-014-0446-7

**Published:** 2014-08-23

**Authors:** Eva C. Bonefeld-Jørgensen, Manhai Long, Stine Overvad Fredslund, Rossana Bossi, Jørn Olsen

**Affiliations:** 1 Department of Public Health, Centre for Arctic Health & Cellular and Molecular Toxicology, Aarhus University, Aarhus, Denmark; 2 Department of Environmental Science, Aarhus University, Aarhus, Denmark; 3 Section for Epidemiology, Department of Public Health, Aarhus University, Aarhus, Denmark

**Keywords:** Breast cancer, Perfluoroalkyl substances, Premenopausal Danish women, Prospective collected data

## Abstract

**Objective:**

Animal studies have indicated that perfluoroalkylated substances (PFAS) increase mammary fibroadenomas. A recent case–control study in Greenlandic Inuit women showed an association between the PFAS serum levels and breast cancer (BC) risk. The present study evaluates the association between serum levels of PFAS in pregnant Danish women and the risk of premenopausal BC during a follow-up period of 10–15 years using prospectively collected exposure data during the pregnancy.

**Methods:**

Questionnaire and blood samples were taken during 1996–2002 and at the end of follow-up, all 250 BC cases and 233 frequency-matched controls were chosen for further analyses. Serum levels of ten perfluorocarboxylated acids, five perfluorosulfonated acids, and one sulfonamide (perflurooctane-sulfonamide, PFOSA) were determined by liquid chromatography-tandem mass spectrometry with electrospray ionization in negative mode. Computer-assisted telephone interviews taken during pregnancy provided data on potential confounders.

**Results:**

Weak positive and negative insignificant associations were found between BC risk and levels of perfluorooctane sulfonamide (PFOSA) and perfluorohexanesulfonate (PFHxS), respectively. Grouped into quintile, the BC cases had a significant positive association with PFOSA at the highest quintiles and a negatively association for PFHxS. Sensitivity analyses excluding uncertain cases caused stronger data for PFOSA and weaker for PFHxS. No further significant associations were observed.

**Conclusions:**

This study does not provide convincing evidence for a causal link between PFAS exposures and premenopausal BC risks 10–15 years later.

**Electronic supplementary material:**

The online version of this article (doi:10.1007/s10552-014-0446-7) contains supplementary material, which is available to authorized users.

## Introduction

The incidence of breast cancer (BC) has been increasing worldwide in the last decades and BC accounts for 23 % of the total cancer cases and 14 % of cancer deaths among females [1]. BC incidence in Denmark has increased in the last six decades [2] to reach 144 cases per 100,000 woman year. Approximately one in ten women will develop BC at some time in their lives [3]. In spite of intense research, the reasons for the increasing BC incidence are only partly known. Research should focus on exposures that have increased over time such as persistent organic pollutants (POPs), e.g., perfluoroalkylated substances (PFAS).

*BRCA1* and *BRCA2* gene mutations account for about 5–10 % of all breast cancers [4]. In addition to age, known risk factors include earlier menarche, later menopause, older age at first childbirth, parity, and short duration of breast feeding, but they explain only a small part of the increasing BC trend. Changes in diet (e.g., high intake of fat), post-menopausal obesity, and alcohol consumption, smoking, and low physical activity may also play a role [5–7].

The risk of BC increases with earlier menarche and later menopause, indicating that breast tissue is sensitive to prolonged endogenous steroid exposure, as seen with exogenous hormone replacement therapy (HRT). Still, all established BC risk factors, including genetic inheritance and factors contributing to lifelong exposure to active estrogens, endogenous and synthetic hormones, can only explain <40 % of all cases [8]. For this reason, we need to identify other potential risk factors. In this study we focus on PFAS, a large group of chemicals used since the 1950s in different industrial and commercial applications (e.g., Teflon, carpets, furniture, foodstuff packing, etc.). These fluorinated chemicals were until recently considered metabolically inert and nontoxic [9]. Available evidence suggests that the transformation or biodegradation of precursor perfluorinated chemicals occurs via both abiotic and biotic degradation pathways where perfluorooctane sulfonate (PFOS) and perfluorooctanoic acid (PFOA) are typical final degradation products [10, 11]. In 2001, it was discovered that PFAS are accumulating in the environment, and in animal and human tissues with a global distribution [10–12].

The PFAS bind to blood proteins and are stored mainly in liver, kidney, and bile excretions [13]. Humans are exposed to PFAS through occupational settings, environmental exposures, and/or through contact with consumer goods (e.g., diet, air, water, food, and household dust) where PFAS have been found.

The PFAS include the perfluorocarboxylated acids (PFCAs) and perfluorosulfonated acids (PFSAs), which include PFOA and PFOS. PFOS and PFOA are the two most studied PFAS because they are found at relative higher levels compared to other PFAS and laboratory procedures in the past were not sensitive enough to identify lower concentrations. Recently, PFOS has been added to Annex B of the Stockholm Convention on POPs [14]. The biological effects of PFAS have been studied in more detail mainly in rodents, and limited data are available for other species and humans [13, 15]. Studies in animals have documented an array of toxicological outcomes including liver hypertrophy and tumors [16], thyroid hormone alterations, decreased serum cholesterol and glucose, developmental toxicity, immunotoxicity, and carcinogenic potency [17, 18]. Animal and in vitro studies have also suggested that PFAS may have potential geno- and neuro-toxic effects [19, 20]. The US EPA has proposed PFOA to be classified as a rodent carcinogen with relevance to humans [21]. A rat study [22] reported a statistically significant increase in mammary fibroadenomas and Leydig cell adenomas, whereas two other rat studies did not find increased incidence of mammary-gland neoplasms upon a 2-year chronic dietary administration of ammonium perfluorooctanoate [23, 24]. Thus, conflicting data for PFOA exposure in rats are reported. In mice, however, gestational exposure to PFOA compared to non-exposed controls was found associated with altered mammary-gland development in dams and female offspring, and a significant reduction in mammary differentiation among exposed dams was evident also affecting the epithelial involution and altered milk protein gene expression [25]. Because of these data, the US EPA Science Advisory Board recommended to reconsider the possible impact of PFOA on mammary tissues [21, 26].

An association between PFAS serum levels and the risk of BC was recently reported for the first time in a small case–control study from Greenland [7], and it was found that the genetic polymorphisms in *CYP1A1* (Val) and *CYP17* (A1) may increase the BC risk among Inuit women, and that the risk increases with higher serum levels of PFOS and PFOA [27].

We now report results from a much larger study within a well-defined cohort, namely the Danish National Birth Cohort (DNBC). We estimate the association between concentrations of PFAS determined during pregnancy, and the risk of BC during a follow-up period of 10–15 years postpartum.

## Methods

### Study design and population

The study was designed as a case-cohort study nested within the Danish National Birth Cohort (DNBC), which was established from 1996 to 2002 and include about 100,000 pregnancies. About half of all pregnant women in Denmark in this time period were invited by their general practitioners (GPs) to take part in the study and about 60 % accepted this invitation.

Questionnaires on lifestyle and environmental exposure (including diet, height, weight, diseases in the family, smoking, and alcohol intake) were administered by a computer-administered interview (CAT) by trained female interviewers twice during pregnancy and 6 months postpartum [28, 29]. All questionnaires are available in an English translation at www.DNBC.dk. Blood was drawn from the mother in the first and second trimester during pregnancy and from the umbilical cord taken shortly after birth and stored at −80 °C. The DNBC participant was followed up to 2010 in this study.

For the present study, all 250 women diagnosed with breast cancer after recruitment according to the cancer registry (BC) and 233 controls (frequency matched on age and parity; taken at random from the entire cohort at baseline) were selected for further PFAS analyses. The information on the BC diagnosis was taken from the National Patient Registry [30]. Neither the sample size nor previous findings justified sub-classification of BC. In our sampling, the BC cases were classified as given by their ICD codes (DC500, 500B, 501, 502, 503, 504, 505, 508, and 509). Blood was drawn between the 6th and 14th gestation weeks for analyses of PFAS.

### PFAS measurements

Ten perfluorocarboxylated acids (PFCAs, C5–C14): perfluoro-*n*-pentanoic acid (PFPeA), perfluorohexanoic acid (PFHxA), perfluoroheptanoic acid (PFHpA), PFOA, perfluorononanoic acid (PFNA), perfluorodecanoic acid (PFDA), perfluoroundecanoic acid (PFUnA), perfluorododecanoic acid (PFDoA), perfluorotridecanoic acid (PFTrA), perfluorotetradecanoic acid (PFTeA); five perfluoroalkyl-sulfonates [PFSAs, C4–C10: perfluorobutane sulfonate (PFBS), perfluorohexane sulfonate (PFHxS), perfluoroheptane sulfonate (PFHpS), PFOS and perfluorodecane sulfonate (PFDS)] and one sulfonamide (perflurooctane-sulfonamide, PFOSA), were determined at the Department of Environmental Science, Aarhus University. The extraction method was based on solid phase extraction (SPE) as described by Keller et al. [31]. Before extraction, the samples were spiked with ^13^C-labelled internal standards (^13^C_2_-PFHxA, ^13^C_4_-PFOA, ^13^C_5_-PFNA, ^13^C_2_-PFDA, ^13^C_2_-PFUnA, ^13^C_2_-PFDoA, ^13^C_8_-PFOSA, ^18^O_2_-PFHxS, and ^13^C_4_-PFOS). Instrumental analysis was performed by liquid chromatography–tandem mass spectrometry (LC–MS–MS) with electrospray ionization (ESI) in negative mode. The samples were extracted and analyzed in batches together with a procedural blank and two control samples, represented by an aliquot of test material previously analyzed in a ring test and for which assigned values for PFOS and PFOA concentrations are available. Detection limits ranged from 0.02 to 0.7 ng/ml. Quality control: method performance is regularly tested three times a year by participating in the Arctic Monitoring and Assessment Programme (AMAP) Ring Test for Organic Pollutants in Human Serum organized by Institute Nationale de Santé Publique du Québec [32]. The information about the performance of the method (detection limits, precision, and bias) and the results from the ring test are given in Online Resource Table 1S and 2S, and Figure 1S.

### Statistical analysis

The PFAS concentrations were grouped into ΣPFSA (sum of PFBS, PFHxS, PFHpS, PFOS, PFDS, and PFOSA) and ΣPFCA (sum of PFPeA, PFHxA, PFHpA, PFOA, PFNA, PFDA, PFUnA, PFDoA, PFTrA, and PFTeA). In addition, analyses were performed for the single compounds detected in all samples including the five PFAS; PFOS, PFHxS, PFOA, PFNA, and PFOSA. PFOSA was also analyzed alone because it is chemically different from the other PFSA, being an amide and not an acid, and because PFOSA and other sulfonamides are precursors to the corresponding PFOS. For all PFAS, the detection limits were calculated, and the exact given concentration was used if the value was above this limit. If the value was below the detection limit, we assigned a value given by the detection limit divided by two.

The distribution of data was checked by Q–Q plots. The natural logarithmic transformed variables made the distribution more symmetrical and thus the analysis was performed on the ln-transformed data. The PFAS levels were analyzed as continuous variables and in a priori-decided quintiles. The correlation of PFAS and potential confounders was analyzed using Pearson correlation analyses. Potential confounders considered for this analysis included age at blood drawing, BMI before pregnancy, total number of gravidities, oral contraceptives (OC) use, age of menarche, smoking status, alcohol intake, maternal education, and physical activity based on a priori well-established breast cancer risk factors.

Although all the cases were eligible for being sampled as controls at the baseline of follow-up, none were in the sampled set of controls and since we have almost complete follow-up, unconditional logistic regression models were used to estimate the relative risks (RRs) and 95 % confidence intervals (95 % CI). The PFAS concentrations were included as categorical and continuous variables into the model together with potential confounders. The change in estimate principle [33] was used to identify potential confounders. However, changes in estimate of more than 5 % were only observed for BMI and menarche age for PFNA and BC risk. For the other PFAS, the estimate change was <5 %. Thus, the results of RRs are given as crude and adjusted RR by including all potential confounders mentioned above. Since age at cancer occurrence influence the risk profile, RR was also estimated by stratification of cancer patient by at onset ≤40 and >40.

All statistical analysis was performed using SPSS version 17.0 (SPSS Inc. Chicago, IL, USA). The statistically significant level was set at 5 %.

### Ethical approval

The study was approved by The Danish Data Protection Agency on the 15th of March 2011 and by The Danish National Committee on Biomedical Research Ethics on the 24th of March 2011.

## Results

### Lifestyle and reproductive characteristics of the study population

Table 1 shows that the age at interview and blood sampling were similar for cases and controls with a mean age of 40 at diagnosis. The controls had a slightly higher BMI (*p* = 0.02). No further larger differences of the characteristics between cases and controls were observed (Table 1).

**Table 1:** Data on breast cancer cases and their controls

Characteristics	Case			Control			*p*
	*n*	Mean	Min–max	*n*	Mean	Min–max	
Age at interview (years)	250	29.8	19–42	233	29.7	20–41	0.77
Age at blood sampling (years)	250	30.4	21–42	233	29.6	20–42	**0.03**
Age at diagnosis (years)^a^	250	40.8	31–53	233	40.3	30–52	0.82
BMI before pregnancy (kg/m^2^)	241	22.9	16.9–38.7	231	23.8	16.3–43.4	**0.02**
BMI at interview (kg/m^2^)	237	24.0	18.3–38.9	221	25.0	18.1–42.3	**0.02**
Number of gravidities	250	0.5	0–6.0	233	0.4	0–5.0	0.71
Menarche age (years)	229	13.2	9.0–19.0	217	13.3	10.0–18.0	0.23
Beer (glass/week)	226	0.1	0–2.0	217	0.1	0–2.0	0.26
Wine (glass/week)	207	0.6	0–7.0	195	0.5	0–4.0	0.36
Physical activity (h/day)	250	5.7	0–53.3	233	5.6	0–51.7	0.42
OC use (yes %)	250	*24.4 %*		233	*24.9 %*		0.90
Smoking (yes %)	250	*27.6 %*		233	*24.0 %*		0.37
TTP	219			217			0.10
Did not try to get pregnant (%)	4	*1.8 %*		8	*3.7 %*		
Got pregnant immediately (%)	32	*14.6 %*		39	*18.0 %*		
1–2 months (%)	37	*16.9 %*		42	*19.4 %*		
3–5 months (%)	54	*24.7 %*		31	*14.3 %*		
6–12 months (%)	39	*17.8 %*		36	*16.6 %*		
>12 months (%)	53	*24.2 %*		61	*28.1 %*		
Education	249			233			0.78
Long/medium (%)	150	*62.8 %*		152	*65.2 %*		
Short (%)	82	*32.8 %*		67	*28.8 %*		
Unskilled (%)	17	*6.8 %*		14	*6.0 %*		

Statistics: Independent *t* test was used to compare the graphical continuous variables (age, BMI, pregnancies, TTP, menarche age, alcohol intake (wine and beer), and maternal physical activity. Pearson’s Chi-square test was used to check the difference between cases and controls for OC use, smoking, maternal education status. The non-italics are continuous data and italics are category variables given in %.

Significant *p* values (<0.05) are given in* bold*

*BMI* body mass index, *OC* oral contraceptive use, *TTP* time to pregnancy

^a^The age at diagnosis is given for the cases and for the corresponding controls it is defined as the age for the matched controls

### BC and serum levels of perfluoroalkyl substances (PFAS)

Five PFAS (PFOS, PFHxS, PFOA, PFNA, and PFOSA) were detected in all samples while for the remaining 11 PFAS the levels were below the detection limit in 0.2–99.4 % of the samples (Online Resource Table 3S).

The data for the 16 determined PFAS, including ten PFCAs (C5–C14), five PFSAs (C4–C10) and PFOSA were analyzed for its association with potential confounders in controls. Weak positive correlations were found between some PFAS and BMI, smoking, alcohol, and maternal education. A negative correlation was found for gravidities.

The mean serum levels for the analyzed five single compounds were for controls as follows: PFOS (30.6 ng/ml), PFOA (5.2 ng/ml), PFHxS (1.2 ng/ml), PFNA (0.5 ng/ml), and PFOSA (3.5 ng/ml), respectively.

The following significant correlation coefficients were found between PFOS versus PFOA (0.69), versus PFOSA (0.58), versus PFNA (0.42), and PFHxS (0.15). Other significant correlation coefficients were observed for PFOSA versus PFOA (0.36), PFNA versus PFOA (0.46) and versus PFHxS (0.29), and PFHxS versus PFOA (0.17). Knowing that PFOSA is a precursor for PFOS can partly explain their relatively high correlation coefficient, whereas the correlation coefficient of 0.36 for PFOSA versus PFOA might suggest common sources of exposure.

For the crude and adjusted data, a weak positive association was found between BC risk and the continuous PFOSA data (unadjusted RR 1.03, 95 % CI 1.00–1.07; adjusted RR 1.04, 95 % CI 0.99–1.08), whereas negative relations were seen for PFHxS (unadjusted 0.66, 95 % CI 0.47–0.92; adjusted RR 0.66, 95 % CI 0.47–0.94). No further significant association between the other continuous PFAS serum data and the RRs of the BC were observed (Table 2).

**Table 2:** Relative risk (RR) for breast cancer according to PFAS exposure

PFAS (ng/ml)	Crude		Adjusted^a^	
	Cases/controls	RR	Cases/controls	RR (95 % CI)
PFOS	250/233	0.99^b^	221/215	0.99 (0.98–1.01)^b^
<20.42	48/49	1.0 (ref)	42/46	1.0 (ref)
20.42–25.31	55/42	1.34	52/40	1.51 (0.81–2.71)
25.31–30.20	56/41	1.39	49/39	1.51 (0.82–2.84)
30.20–39.07	47/50	0.96	43/44	1.13 (0.59–2.04)
>39.07	44/51	0.88	35/46	0.90 (0.47–1.70)
PFOA	250/233	0.99^b^	221/215	1.00 (0.90–1.11)^b^
<3.69	51/46	1.0 (ref)	46/43	1.0 (ref)
3.69–4.59	49/49	0.90	46/48	0.97 (0.53–1.75)
4.59–5.42	50/47	0.96	43/41	1.02 (0.56–1.89)
5.42–6.53	53/42	1.14	46/40	1.14 (0.62–2.12)
>6.53	47/49	0.87	40/43	0.94 (0.51–1.76)
PFNA	250/233	1.00^b^	221/215	0.76 (0.30–1.94)^b^
<0.32	49/48	1.0 (ref)	44/41	1.0 (ref)
0.32–0.42	53/44	1.14	50/40	1.10 (0.60–2.02)
0.42–0.50	58/39	0.74	38/46	0.75 (0.41–1.40)
0.50–0.64	45/52	1.12	47/40	1.08 (0.58–1.99)
>0.64	45/50	0.78	42/48	0.80 (0.43–1.47)
PFHxS	250/233	**0.66** ^**b**^	221/215	**0.66 (0.47–0.94)** ^b^
<0.76	64/38	1.0 (ref)	58/35	1.0 (ref)
0.76–0.92	49/46	0.63	42/42	0.64 (0.34–1.18)
0.92–1.12	52/46	0.67	47/43	0.70 (0.38–1.29)
1.12–1.35	37/56	**0.39**	32/52	**0.38 (0.20–0.70)**
>1.35	48/47	0.61	42/43	0.61 (0.33–1.12)
PFOSA	250/233	**1.03** ^b^	221/215	1.04 (0.99–1.08)^b^
<0.93	48/49	1.0 (ref)	43/47	1.0 (ref)
0.93–1.70	53/44	1.23	48/41	1.38 (0.75–2.52)
1.70–2.83	44/53	0.85	38/49	0.91 (0.49–1.66)
2.83–5.75	46/50	0.94	41/45	1.11 (0.60–2.05)
>5.75	59/37	1.63	51/33	**1.89 (1.01–3.54)**
sumPFSA	250/233	1.00^b^	221/215	1.00 (0.99–1.01)^b^
<24.43	52/45	1.0 (ref)	46/44	1.0 (ref)
24.43–29.58	52/46	0.96	48/42	1.19 (0.66–2.16)
29.58–35.67	53/42	1.09	38/42	1.15 (0.62–2.13)
35.67–45.38	49/48	0.88	41/41	1.09 (0.59–2.02)
>45.38	44/51	0.75	51/46	0.82 (0.43–1.55)
sumPFCA	250/233	0.99^b^	221/215	1.00 (0.91–1.09)^b^
<4.82	50/47	1.0 (ref)	45/43	1.0 (ref)
4.82–5.85	49/48	0.96	44/47	0.87 (0.48–1.59)
5.85–6.80	49/48	0.96	43/44	0.92 (0.50–1.69)
6.80–8.02	57/38	1.44	52/34	1.49 (0.80–2.77)
>8.02	44/52	0.80	37/47	0.76 (0.41–1.41)
sumPFAS	250/233	1.00^b^	221/215	1.00 (0.99–1.01)^*b*^
<29.54	50/46	1.0 (ref)	44/45	1.0 (ref)
29.54–35.32	53/48	1.00	50/44	1.24 (0.68–2.25)
35.32–42.17	56/37	1.39	49/37	1.41 (0.76–2.63)
42.17–53.11	45/51	0.80	40/45	0.96 (0.52–1.78)
>53.11	46/49	0.86	38/44	0.94 (0.50–1.78)

Bold data indicates significance (*p* < 0.05).

^a^Adjusted for age at blood sampling, BMI before pregnancy, gravidity, OC use, menarche age, smoking during pregnancy, alcohol intake, maternal education and physical activity

^b^RR was obtained from the original continuous variables.

The logistic regression analyses of the quintiles [using the 1st (lowest) quintile as the reference] showed a significant elevated BC risk for PFOSA in the 5th quintile with a RR of 1.89 (95 % CI 1.01–3.54) upon adjustment for confounders (Table 2). For the PFHxS, a significant negative association to BC was observed for the 4th quintile versus 1st quintile [RR 0.38 (95 % CI 0.20–0.70)] (Table 2). Upon stratification for age at diagnosis, the associations for both compounds were further strengthened (Table 3); for the continuous adjusted PFOSA data the RR was 1.07 (95 % CI 1.00–1.14) and for PFOSA in the highest quintile (RR 2.45, 95 % CI 1.00–6.00) among women 40 years of age or less. For PFHxS, the significance of the continuous data disappeared, but the risk was significant in all quintiles but the third (RR range, 0.30–0.41). After stratification for BC occurrence at >40 years of age, significances disappeared (Table 3).

**Table 3:** Adjusted relative risk (RR) of breast cancer in accordance to PFAS exposure stratified by age at diagnosis

PFAS (ng/ml)	Age <40		Age >40	
	Cases/controls	RR (95 % CI)	Cases/controls	RR (95 % CI)
PFOS	132/120	0.99 (0.97–1.02)^a^	118/113	1.00 (0.98–1.02)^a^
<20.42	26/22	1.00 (ref)	22/27	1.00 (ref)
20.42–25.31	28/23	1.22 (0.52–2.88)	27/19	2.30 (0.94–5.64)
25.31–30.20	30/20	1.38 (0.58–3.30)	26/21	1.90 (0.73–4.97)
30.20–39.07	22/30	0.79 (0.33–1.88)	25/20	2.22 (0.87–5.69)
>39.07	26/25	1.01 (0.41–2.50)	18/26	0.88 (0.33–2.38)
PFOA	132/120	0.99 (0.84–1.18)^a^	118/113	1.01 (0.88–1.16)^a^
<3.69	26/19	1.00 (ref)	25/27	1.00 (ref)
3.69–4.59	24/31	0.66 (0.28–1.55)	25/18	1.77 (0.73–4.31)
4.59–5.42	29/19	1.26 (0.51–3.15)	21/28	0.93 (0.38–2.27)
5.42–6.53	28/25	0.83 (0.35–1.99)	25/17	1.91 (0.76–4.83)
>6.53	25/26	0.77 (0.31–1.91)	22/23	1.17 (0.48–2.86)
PFNA	132/120	0.54 (0.13–2.32)^a^	118/113	1.09 (0.30–3.96)^a^
<0.32	29/19	1.00 (ref)	20/24	1.00 (ref)
0.32–0.42	33/22	1.05 (0.44–2.50)	24/22	1.19 (0.48–2.96)
0.42–0.50	21/27	0.52 (0.22–1.26)	22/24	1.30 (0.52–3.25)
0.50–0.64	28/28	0.82 (0.35–1.88)	27/15	1.85 (0.70–4.86)
>0.64	21/24	0.56 (0.23–1.37)	25/28	1.14 (0.46–2.82)
PFHxS	132/120	0.64 (0.39–1.05)^a^	118/113	0.71 (0.43–1.15)^a^
<0.76	40/19	1.00 (ref)	24/19	1.00 (ref)
0.76–0.92	29/29	**0.39 (0.17–0.88)**	20/17	1.23 (0.44–3.42)
0.92–1.12	23/21	0.56 (0.23–1.35)	29/25	1.04 (0.40–2.68)
1.12–1.35	19/26	**0.30 (0.12–0.72)**	18/30	0.52 (0.20–1.35)
>1.35	21/25	**0.41 (0.17–0.96)**	27/22	1.01 (0.40–2.54)
PFOSA	132/120	**1.07 (1.00–1.14)** ^a^	118/113	1.01 (0.97–1.07)^a^
<0.93	29/33	1.00 (ref)	19/16	1.00 (ref)
0.93–1.70	32/27	1.53 (0.70–3.32)	21/17	1.30 (0.48–3.56)
1.70–2.83	22/24	1.04 (0.45–2.40)	22/29	0.96 (0.37–2.51)
2.83–5.75	22/24	1.10 (0.46–2.59)	24/26	1.37 (0.52–3.61)
>5.75	27/12	**2.45 (1.00–6.00)**	32/25	1.62 (0.61–4.29)
sumPFSA	132/120	1.00 (0.98–1.02)^a^	118/113	1.00 (0.98–1.02)^a^
<24.43	31/23	1.00 (ref)	21/22	1.00 (ref)
24.43–29.58	28/26	0.90 (0.40–2.03)	24/21	2.00 (0.67–5.28)
29.58–35.67	24/18	1.06 (0.44–2.52)	29/24	1.81 (0.38–3.84)
35.67–45.38	23/30	0.62 (0.27–1.44)	26/18	2.62 (0.44–5.19)
>45.38	26/23	1.01 (0.42–2.40)	18/28	0.79 (0.29–2.14)
sumPFCA	132/120	0.99 (0.85–1.15)^a^	118/113	1.00 (0.89–1.14)^a^
<4.82	26/23	1.00 (ref)	24/24	1.00 (ref)
4.82–5.85	27/23	1.04 (0.44–2.46)	22/25	0.98 (0.39–2.40)
5.85–6.80	27/27	0.90 (0.38–2.12)	22/21	1.25 (0.49–3.22)
6.80–8.02	30/20	1.37 (0.57–3.27)	28/18	1.73 (0.69–4.34)
>8.02	22/27	0.68 (0.28–1.65)	22/25	1.01 (0.40–2.51)
sumPFAS	132/120	1.00 (0.99–1.02)^a^	118/113	1.00 (0.99–1.02)^a^
<29.54	29/24	1.00 (ref)	21/22	1.00 (ref)
29.54–35.32	31/25	1.22 (0.54–2.78)	22/24	1.67 (0.66–4.21)
35.32–42.17	23/16	1.09 (0.44–2.69)	33/21	2.50 (0.96–6.52)
42.17–53.11	22/32	0.64 (0.27–1.49)	23/20	2.09 (0.78–5.61)
>53.11	27/23	1.17 (0.49–2.79)	19/26	0.94 (0.34–2.56)

Adjusted for age at sampling, BMI before pregnancy, gravidity, OC use, menarche age, smoking during pregnancy, alcohol intake, maternal education and physical activity. Bold data indicates significance (*p* < 0.05).

^a^RR obtained from the original continuous variables.

Recently, 72 of our breast cancer cases were withdrawn from the current Danish National Patient Register (DNPR) making the BC cancer status uncertain for this group. We therefore repeated all our analyses after excluding these cases and the following minor differences were observed. The BMI association disappeared. In the analyses based on continuous data, PFOSA was slightly more strongly correlated with BC risk (unadjusted RR 1.04, 95 % CI 1.00–1.08, adjusted RR 1.05, 95 % CI 1.00–1.09), and again a negative correlation of PFHxS and BC risk was found for the unadjusted data (RR 0.65, 95 % CI 0.44–0.96), whereas upon adjustment, the significant correlation disappeared (RR 0.67, 95 % CI 0.45–1.01). The logistic regression analyses of the quintiles also showed a stronger significant correlation for PFOSA in the 5th quintile (adjusted RR 2.40, 95 % CI 1.20–4.83). For PFHxS, a significant negative correlation with BC risk was observed in the 4th quintile before and after adjustment of confounders. Upon stratification for age at diagnosis, the positive association of PFOSA in the highest quintile and BC risk was even stronger for the women younger or 40 years of age (adjusted RR 3.42, 95 % CI 1.25–9.36). The negative correlation of PFHxS and BC risk was only observed at the 2nd and 4th quintile (adjusted RR 0.3). Among women older than 40 years of age, no significant correlation with BC risk was observed for any of the PFAS congeners. In addition, the levels of PFOSA in cases were significantly higher than the controls (*p* = 0.04).

## Discussion and conclusions

The results of this study suggest no strong or coherent association between BC occurrence and the measured PFAS concentrations in plasma taken up to 15 years before the diagnosis of breast cancer. The PFOSA association was weak for the continuous data and statistical significance was restricted to the highest exposure level with no support by the estimate for trends. The few positive association could be chance findings related to multiple testing. However, sensitivity analyses excluding 72 cases recently withdrawn from the DNPR resulted in stronger associations between PFOSA and RR of BC and weaker for the PFHxS data. Moreover, it should be noted that a similar association with PFOSA serum levels was found in our previous study on breast cancer in Greenlandic women [7]. In contrast to the present study, the study on BC in Greenlandic women was based on the PFAS serum levels taken at the time of diagnosis and included also post-menopausal older women (median age 50–54 vs. this study 30 years at blood drawing) [7].

We studied BC risk using prospectively collected BC data in predominately premenopausal women. We have exposure data from early in pregnancy, which probably covers the exposure status for a time period that at least overlaps with the causal time window of interest. Our study has a size that leaves room for some statistical uncertainty, as indicated by the wide confidence limits, but if there is a linear dose–response association, the trend test would probably pick it up. There are some limitations of this study related to the degree of heterogeneity of the cases, which may have compromised the power of the study if the exposure is only causal for subtypes of BC. We do not have information on case characteristics regarding, i.e., tumor size, nodal status, in situ versus invasive, and immune histochemical markers, i.e. ER, PR, or HER2/neu nor the family history of breast cancer. The blood was drawn between the 6th and 14th gestation week at the GPs at their antenatal care regular visit. Our analyses show rather stable PFAS values over gestational time, and the results are adjusted for gestation age of blood drawing.

For the Greenlandic BC women, the PFOA, PFOS, PFOSA, and PFHxS serum levels were significantly higher in cases and showed a significantly elevated risk for BC according to adjusted data for PFOS [OR 1.03 (1.00; 1.07)], PFOSA [OR 6.13 (1.12–33.64)], and PFHxS [OR 1.40 (0.95–2.05)] ([7], the latter two compounds OR unpublished). In comparison, the PFOS mean levels were slightly higher in Inuit controls than in Danish controls (38.5 vs. 30.6 ng/ml). For PFOSA, the serum mean level was nine times higher in Danish controls than Inuit controls, 3.5 versus 0.4 ng/ml, respectively, and the mean PFHxS serum levels were almost 2.3 times higher in Inuit controls compared to Danish controls, 2.8 versus 1.2 ng/ml, respectively.

It is important to note that data for both PFOSA and PFHxS levels correlate with BC risk in both Inuit and Danish women, although negatively for PFHxS in Danish women. Reverse causation may account for the findings seen in the study from Greenland but this problem is unlikely in the Danish data.

PFOSA is a synthetic fluorocarbon compound used to repel grease and water in food packing along with other consumer applications [34]. It breaks down to PFOS [35]. In addition, PFOSA is a metabolic by-product of *N*-alkylated sulphonamides and *N*-methyl sulphonamidoethnanol primarily used on carpets and textiles [36]. Moreover, PFOSA is thought to be the biological active form of the insecticide sulfluramid by inducing mitochondrial dysfunction [37]. Biomonitoring studies have found PFOSA in livers of wildlife at higher parts per billion and human serum at lower part per billion levels [38–41]. In whole blood (WB), the concentration of PFOSA is about five times higher than in plasma/serum (PLS). Levels up to 19.7–22.9 ng/ml in WB have been reported [42] but the general WB/PLS level was found to be in the range of 3–4.1/0.10–0.6 ng/ml [43–45]. Because of the relatively low level found in WB and unequal distribution to PLS, it makes it uncertain to quantify PFOSA in plasma or serum only. Therefore both media should be measured to assess the real PFOSA blood concentration in future studies. PFOSA was reported to be a potent mitochondrial toxicant [37] and precursor of PFOS, found in human tissues. In addition, PFOSA was the most toxic perfluorinated compound in a study on developmental neurotoxicity of PFAS using the neural transforms cell line PC12 [46].

PFHxS is found in a variety of industrial, consumer, and dietary sources including house dust [47]. In our recent BC study in Inuit, the PFHxS [7] was found to be a potential BC risk factor, whereas in the present study a negative correlation with BC risk was found. However, it should be taken into consideration that the PFHxS serum levels were 2.3 and 4 times higher in Inuit controls and cases, respectively, compared to DK controls and cases, respectively. In addition to the possible differences in exposures to PFHxS between Inuit and Danish women, the timing according to case status should also be considered. If the disease moves perfluorinated compounds to the bloodstream, the risk estimate is biased towards higher values.

No health effects of PFHxS have yet been documented, however PFHxS (C6) share many of the common physical and chemical properties with PFOS (C8), suggesting that PFHxS may also have biological effects similar to PFOS. To our knowledge, no carcinogenicity in relation to PFHxS has previously been reported, but neonatal exposure to PFHxS was reported to cause behavioral and cognitive disturbance in adult mice [48], being similar to observations following PFOS exposure [49]. Moreover, epidemiological studies have reported a positive association between serum levels of PFHxS and attention deficiency/hyperactivity disorder (ADHD) and increased impulsivity in children [50, 51].

Although perfluorooctanesulfonyl fluoride-based chemistry was phased out by 3M in the USA during 2000–2002, the production is continuing and being increased in China [52].

PFAS have been associated with effects being of potential relevance for risk of BC such as (1) hormone disruption in vitro [53–56], (2) genotoxic potentials in vitro [57–60], (3) tumor promoting in rodents including reduction in mammary differentiation and increase in mammary fibroadenomas [21, 25, 26], (4) BC risk factor in Inuit women [7], and (5) immunosuppressive effects in humans [61, 62]. These observations need to be further scrutinized in studies on cellular, toxicological mechanisms involved in vivo*/*ex vivo using exposure of single compounds as well as mixtures reflecting the actual complex mixtures found in human serum.

In conclusion, this study does not document PFAS as overall causes of BC in Danish premenopausal women but does not rule out that such an association may exist and more studies are needed.

## Electronic supplementary material

Below is the link to the electronic supplementary material.

Supplementary material 1 (DOCX 31 kb)
