# Supplementary material for: Breast cancer risk after exposure to perfluorinated compounds in Danish women: a case–control study nested in the Danish National Birth Cohort
Source: Cancer Causes Control. 2014 Aug 23;25(11):1439–48. doi: 10.1007/s10552-014-0446-7 (PMC4215104; doi:10.1007/s10552-014-0446-7)
Supplement: Supplementary file 1 — Supplementary material 1 (DOCX 31 kb) [file 10552_2014_446_MOESM1_ESM.docx]

**Supplementary Material**

**Breast cancer risk after exposure to perfluorinated compounds in Danish Women: A case-control study nested in the Danish National Birth Cohort**

Eva C. Bonefeld-Jørgensen^*#^, Manhai Long^*^, Stine Overvad Fredslund^*^, Rossana Bossi^¤^, Jørn Olsen^**^

^*^Centre for Arctic Health & Cellular and Molecular Toxicology, Department of Public Health, Aarhus University, Denmark, ^¤^ Department of Environmental Science, Aarhus University, Denmark, ** Section for Epidemiology, Department of Public Health, Aarhus University, Denmark

#: Corresponding author

Eva C. Bonefeld-Jørgensen,

[ebj@mil.au.dk](mailto:ebj@mil.au.dk) ,

Phone +45-871 68012; fax +45-8716730

***Validation of the analytical method***

Detection limit and precision of the analytical method for PFAS were determined using spiked rabbit serum (Sigma Aldrich).

The method detection limit (MDL) was calculated as:

MDL = 3 x *s*

Where *s* is the standard deviation for repeated analyses (n=10) of laboratory blank (for those compounds found in the blank) or blank samples spiked to a concentration of 0.5 ng/ml for each compound.

The precision of the method (intra day and inter day) has been calculated at two different concentrations (1 ng/ml and 10 ng/ml).

The results for detection limit and precision of the method are summarized in Table 1S.

Table 1S. Method detection limits (MDL)

| **Compound** | **MDL (ng/ml)** | **Precision (RSD%)**  **1 ng/ml spike** | **Precision (RSD%)**  **10 ng/ml spike** |
| --- | --- | --- | --- |
| PFBS | 0.02 | 6.3 | 11.3 |
| PFHxS | 0.04 | 3.5 | 1.2 |
| PFHpS | 0.05 | 2.5 | 2.7 |
| PFOS | 0.41 | 7.7 | 2.3 |
| PFDS | 0.12 | 3.6 | 8.2 |
| PFOSA | 0.40 | 3.2 | 5.4 |
| PFPeA | 0.10 | 6.7 | 2.9 |
| PFHxA | 0.17 | 3.2 | 1.2 |
| PFHpA | 0.02 | 6.4 | 1.9 |
| PFOA | 0.07 | 2.3 | 2.0 |
| PFNA | 0.09 | 3.5 | 2.3 |
| PFDA | 0.07 | 4.9 | 3.1 |
| PFUnA | 0.25 | 5.3 | 5.1 |
| PFDoA | 0.14 | 5.5 | 8.0 |

The bias of the method (only for PFOS and PFOA) was calculated from repeated analyses (n=x) of human serum from one of the round of the AMAP Ring Test for Persistent Organic Pollutants in Human Serum organized by the Institute Nationale de Santé Publique du Québec, Canada. The values assigned for PFOS and PFOA by the organizer of the Ring test have been used as certified values of the material used to determine the bias of the method. Other PFAS have not been included in the results of the Ring Test before 2013.

Table 2S. Bias of the method (only PFOS and PFOA)

| **Compound** | **Average concentration**  **(± stdv) ng/ml** | **Assigned value (ng/ml)** | **RSD % of the assigned value** | **RSD% from the assigned value** |
| --- | --- | --- | --- | --- |
| PFOA | 8.13 (± 0.29) | 8.08 | 0.985 | 0.7 |
| PFOS | 36.6 (± 2.5) | 34.7 | 1.96 | 5.6 |

***Results from participation in AMAP Ring Test***

The performance of the laboratories participating in the ring tests is evaluated by assigning a z-score to each measured analyte. Z-score is a simple way to evaluate the results in the relation to the uncertainty of the intercalibration. Z-scores between -2 and 2 is regarded satisfactory; z-scores from -3 to -2 and 2 to 3 is regarded as questionable results; z-scores below -3 and higher as 3 is regarded as not acceptable.

The z-scores obtained by our laboratory in the Ring Tests performed from 2010 to 2013 (3 times/year) are summarized in Figure 1S. The concentrations of the analytes are different in the triplicates from each test and ranged from 4.0 to 148 ng/ml for PFOS and from 0.97 to 26.5 ng/ml for PFOA.

Figure 1S. z-scores for PFOS and PFOA obtained in the period 2010-2013.

Table 3S. The percentage of PFAS serum level over and below the methods detection limit (MDL)

| **PFAS** | **N** | **n above MDL** | **% above MDL** | **n below MDL** | **% below MDL** |
| --- | --- | --- | --- | --- | --- |
| PFOS | 484 | 484 | 100 | 0 | 0 |
| PFOSA | 484 | 484 | 100 | 0 | 0 |
| PFHxS | 484 | 484 | 100 | 0 | 0 |
| PFOA | 484 | 484 | 100 | 0 | 0 |
| PFNA | 484 | 484 | 100 | 0 | 0 |
| PFHpS | 484 | 483 | 99.8 | 1 | 0.2 |
| PFDA | 484 | 429 | 88.6 | 55 | 11.4 |
| PFHpA | 484 | 414 | 85.5 | 70 | 14.5 |
| PFDoA | 484 | 289 | 59.7 | 195 | 40.3 |
| PFTrA | 484 | 258 | 53.3 | 226 | 46.7 |
| PFUnA | 484 | 241 | 49.8 | 243 | 50.2 |
| PFDS | 484 | 89 | 18.4 | 395 | 81.6 |
| PFBS | 484 | 22 | 4.5 | 462 | 95.5 |
| PFHxA | 484 | 12 | 2.5 | 472 | 97.5 |
| PFPeA | 484 | 8 | 1.7 | 476 | 98.3 |
| PFTeA | 484 | 3 | 0.6 | 481 | 99.4 |
